# Development of a Paper-Based Analytical Method for the Colorimetric Determination of Calcium in Saliva Samples

**DOI:** 10.3390/s23010198

**Published:** 2022-12-24

**Authors:** Maria Tarara, Paraskevas D. Tzanavaras, George Z. Tsogas

**Affiliations:** Laboratory of Analytical Chemistry, School of Chemistry, Faculty of Sciences, Aristotle University of Thessaloniki, GR-54124 Thessaloniki, Greece

**Keywords:** paper-based analytical devices, colorimetric determination, calcium, methylthymol blue, saliva samples

## Abstract

A novel, rapid, and facile method for the colorimetric determination of calcium using micro-analytical paper-based devices (μ-PADs) was developed. The proposed analytical method utilizes the color differences developing, after the addition of calcium, on the surface of the devices because of the complexation reaction of calcium with Methylthymol Blue (MTB) at room temperature, in alkaline pH. The devices were manufactured with chromatographic paper, using wax barriers, and the analytical protocol was easily implemented without the need of any experimental apparatus except for a simple imaging device. The user must regulate the pH, add the solutions on the paper, and measure the color intensity of the formed Ca(II)–MTB complex with a flatbed scanner. The experimental conditions for optimum color development, the possible interfering substances, and the reliability of the paper devices in different preserving conditions were optimized, with satisfactory results. The method exhibited acceptable detection limits (2.9 mg L^−1^) with sufficiently good precision, which varied from 4.2% (intra-day) to 6.4% (inter-day). Saliva samples from healthy volunteers were successfully analyzed, and the calcium levels were calculated in the range of 30.71 to 84.15 mg L^−1^.

## 1. Introduction

Saliva is an important body fluid, though not the most popular one, because it does not demonstrate the drama of blood, the realism of sweat, and the emotional charge of tears. Saliva is a complex biological fluid consisting of the secretion of three pairs of primary salivary glands and multiple secondary glands [1]. The major salivary glands (parotid, submandibular, and sublingual) are responsible for the secretion of 90% of the 600 mL of saliva produced every day in the human mouth of an average person [2]. Saliva has various functional uses such as (a) oral cavity cleaning, by dissociating bacteria and food particles, thus refreshing the breath, (b) amylase production, an enzyme that, in human saliva, is the catalyst for the hydrolyzation reaction of starch to maltose and occasionally glucose, and (c) the production of certain chemical compounds (lysozyme and SCN^−^), that can destroy various bacteria, rendering saliva essential for the human immune system [2,3]. Saliva consists of approximately 98% water and 2% organic and inorganic substances, such as salivary amylase, mucopolysaccharide, mucin and lysozymes, and some inorganic electrolytes such as sodium, potassium, calcium, magnesium, bicarbonate, chloride, thiocyanate, and phosphate [2,3,4,5].

More than 99% of the calcium in the human body is distributed in the bones and teeth. Calcium is also a threshold nutrient [6]. Below the threshold level, an increasing dietary calcium intake leads to an improved response to calcium. Above the uptake threshold, on the other hand, there is little or no further improvement. This fact complicates the interpretation of many studies of the effects of calcium intake on health, because in some populations, calcium intake exceeds the threshold [7]. In bones, calcium is essential for the structural strength that allows the bone to support the body’s weight and anchor muscles. Calcium differs from most other nutrients in that the body contains a significant store of it, far more abundant than the amount required for short-term needs, but at the same time, this store has a critical structural role. Thus, the effects of calcium deficiency may go unnoticed for a long time, until they manifest as skeletal weakness or fractures [8]. Additionally, the health problems associated with chronic calcium malnutrition are, in the main, slowly developing, and multifactorial conditions that are difficult to trace to any single cause. It is rare for most physicians to see patients with acute pathological conditions of calcium metabolism [8]. Among the inhabitants of the entire planet, the challenges presented by maintaining calcium homeostasis vary widely. Different eating habits can provide quite distinct levels of calcium intake. In the modern lifestyle, the consumption of products with a high calcium content has been drastically reduced. Some of the diseases of modern humans, including brittle bones, hypertension, and colon cancer, may be caused, or at least worsened, by a chronically low dietary calcium intake [9].

One additional disease caused by a low intake of calcium is dental erosion [10]. Calcium and phosphate work in a team as anti-solubilizing agents to regulate the process of tooth demineralization and remineralization [4] and prevent dental erosion. As these ions were found to be reduced in the saliva of patients with active carious lesions [5,11,12], these biochemical parameters play a crucial role in determining the susceptibility of individual caries and other teeth to descaling [13].

The analytical determination of alkaline metal earths such as calcium and magnesium has been studied from the middle of the twentieth century. Analytical methods such as complexometric titrations [14,15], fluorometric titrations [16,17], and spectrophotometric determination [18] have been used since the 1950s. Many instrumental analytical methods have been developed for the determination of calcium in a big variety of samples, utilizing various detection techniques, such as flame atomic absorption spectroscopy (FAAS) [19,20,21], atomic emission spectrometry [22], inductively coupled plasma mass spectrometry (ICP-MS) [23,24,25,26], fluorescence [27,28], ion chromatography [29] UV–Vis spectrophotometry [30], and flow injection [31,32].

A variety of chemical substances has been used from the mid-1970s for the colorimetric determination of Ca(II), including o-cresolphthalein (OCP), [33], dyes of arsenazo-III [34], 1,2-bis(o-aminophenoxy)ethane N,N,N′,N′-tetra-acetic acid acetoxymethyl ester (NM-BAPTA) [35], and methylthymol blue (MTB) [36,37,38].

MTB is a metallochromic indicator containing a thymol group and is used in analytical chemistry as an equivalence point identifier in complex titrations or as a spectrophotometric reagent for the formation of stable metal complexes for the spectrophotometric determination of many metal ions. The molecule of MTB has nine active functional groups, which are distinguished in four carboxylic acids, two phenolic, two amine, and one sulfonyl group, making this chemical compound suitable for the complexation with various metal ions [39]. Depending on the pH of the solution, MTB interacts with many metal ions such as Al^3+^, Bi^3+^, Ca^2+^, Co^2+^, Cu^2+^, Fe^2+^, Fe^3+^, Mg^2+^, Zn^2+^, etc. [40,41,42]. MTB forms stable complexes with Al^3+^ as well as with Zr^4+^, Hf^4+^, and Bi^3+^ under acidic conditions. In contrast, Fe^3+^ reacts with MTB in weakly acidic solutions (pH = 6), while the complexation of alkaline metal earths (Ca^2+^ and Mg^2+^) is carried out in a strong alkaline environment [36,42].

Recently, there is a growing demand for the production and development of simple, inexpensive, and easy-to-implement analytical devices that can be used with minimal resources and provide fast and reliable results. In view of this growing need, paper-based devices are considered highly desirable analytical sensors for portable and low-cost assays that can be used with minimal resource and instrument requirements and provide rapid results [43]. The main advantages of paper as a substrate for analytical applications are the following: (a) it is inexpensive and easily supplied, (b) it has a great proportion of active surface with respect to solution volume, (c) solutions can saturate the devices without the use of pumps and external forces, (d) its fibrous structure enables the storage of reagents, their drying, and their use after a long period of time, and (e) its surface can be modified in such a way as to allow and favor the analytical determination of individual chemical compounds [42]. In addition, these analytical sensors are also portable or easily transported and installed at the point of need without loss of functionality, and analyte detection can be performed using widely available domestic detectors such as smartphones or flatbed scanners [42,44]. Since their introduction as analytical devices in 2007 by the research group of Prof. G. Whitesides [45], many researchers, including our research group, have studied a considerable number of paper-based methods and developed several fully functional paper platforms for the detection of a wide variety of environmental, biochemical, and food samples [42,46,47,48].

In this study, a paper-based analytical method for the determination of calcium, relying on the direct complexation reaction with MTB in a highly basic medium and on the alteration of the color produced in the detection zone, detected by a flatbed scanner, is reported. Our devices are cheap and easily fabricated and converted into hydrophobic sensing zones. Additionally, the detailed protocol can be applied without the use of specialized instrumentation, and the user is not required to have any technical experience. Moreover, the applicability of this method was evaluated for the determination of calcium in human saliva samples, compared with a UV–Vis method, and the results were satisfactory regarding sensitivity, limits of detection and qualification, and reproducibility.

## 2. Materials and Methods

### 2.1. Reagents and Solutions

Methylthymol Blue (MTB), calcium chloride dihydrate, potassium chloride, sodium chloride, sodium sulfite, sodium hydroxide, potassium dihydrogen phosphate, and sodium hydrogen carbonate were provided by Merck (Darmstadt, Germany), while magnesium chloride hexahydrate and nitric acid were provided by Panreac (Madrid, Spain). All chemical substances were of analytical grade, and all solutions were prepared with de-ionized water. The standard stock CaCl_2_ × 2H_2_O solution (1000 mg L^−1^ Ca(II)) was prepared weekly in de-ionized water. Working Ca(II) solutions were prepared daily by the appropriate dilutions of the stock Ca(II) solution. The pH was adjusted with the use of a 2.0 mol L^−1^ NaOH stock solution. MTB working solutions were prepared daily, while the MTB stock solution (25 mmol L^−1^) was prepared on a weekly basis with de-ionized water. Finally, cation and anion stock solutions for the selectivity investigation had a concentration of 500 mmol L^−1^ for each ion studied.

### 2.2. Apparatus

The fabrication of the devices was accomplished by drawing a circular pattern in a white background in PowerPoint and printing it by the deposition of wax on the paper surface (Whatman No. 1 Chromatography paper) with the use of a solid-ink printer (ColorQube 8580DN, Xerox). The influence of the pH in our analytical methodology was determined with a pH-meter (Orion). The photographs of the paper devices were taken using a mobile smartphone (Xiaomi Redmi Note 10) and a flatbed scanner (HP Scanjet 4850).

### 2.3. Fabrication of the Paper Devices

Hydrophilic circular sensing areas were designed with the PowerPoint program. The term device is used herein for the description of a series of individual, round-shaped, sensing zones printed in a row. We drew a circular pattern in a white background in PowerPoint, multiplied it 50 times so that the whole surface of the Whatman paper No 1 was covered by these devices, and printed them on the paper. We needed to establish hydrophobic barriers to enclose the aqueous solutions, and for the devices to be suitable for the analysis, the wax had to penetrate both surfaces of the paper substrate. For this purpose, the devices were heated in a common furnace at 120 ± 5 °C for 2 min to melt the wax ink isolating the sensing area. The devices had a diameter of 0.80 cm and consisted of an internal area (sensing zone) with a diameter of 0.40 cm, isolated by a wax barrier of 0.20 cm thickness (wax zone). For the homogenous deposit and dryness of the reagents, to accomplish repeatable results, we used chromatography paper (Whatman No. 1) because it has high thickness (0.18 mm), thus tearing phenomena would be avoided, as well as significant mass per area (87 g m^−2^), to ensure the possibility of adding maximum possible solution volumes and achieving a quick drying of the devices.

### 2.4. Experimental Process

**Figure 1:**
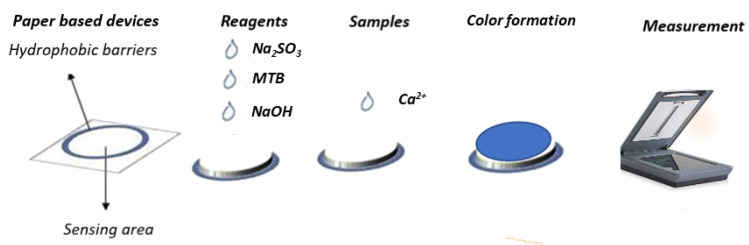
Experimental process for the paper-based analytical method for calcium determination.

The experimental procedure was easy to follow, with no requirements for laboratory instrumentation. In brief, in the center of the sensing zone, we deposited Na_2_SO_3_ (1 μL, 2.5 mmol L^−1^), NaOH (1 μL, 0.2 mol L^−1^), MTB (1 μL, 5 mmol L^−1^). Between each addition, the devices were left to dry at room temperature for 10 min. Finally, 1 μL of the calcium standard solutions or saliva samples were deposited on the surface of each device. After the calcium addition, a blue color formed on the paper surface in dependance of its concentration (from pale to dark blue), and the color intensity was measured by o common flatbed scanner. The images were transferred to a laptop and saved as JPEG files with 300 dpi analysis, and afterwards the mean color intensity was measured with the use of the Image J program through the red channel of the RGB mode (Figure 1).

### 2.5. Real Samples

Random saliva samples were voluntarily donated by male and female members of the laboratory, were collected in Salivette cotton swabs (Sarstedt, Numbrecht-Germany), and were processed immediately. The volunteers did not have anything to drink or eat for at least 30 min before the sample donation. The swab was placed in the mouth and chewed for about 60 s to stimulate salivation. The swab with the absorbed saliva was placed back in the Salivette and was centrifugated for 2 min at 1000× *g*, yielding a clear saliva sample in the conical tube. Due to the selectivity and sensitivity of the proposed method, sample preparation included only the following easy and rapid steps: 2- to 8-fold dilution with de-ionized water depending on the levels of Ca^2+^ in the real samples and analysis by the paper based colorimetric method.

### 2.6. Confirmative UV–Vis Method

A UV–Vis method was used as a corroborative method. The analysis was performed at 600 nm using a JASCO V-530 UV/Vis spectrophotometer and matched quartz cells of 1 cm path length. Phosphate buffer, pH 12 (1 mL, 100 mmol L^−1^), MTB (1 mL, 1 mmol L^−1^), and sample/standards (1 mL) were added in the cell. The stock solution of Ca^2+^ was diluted in HNO_3_ (0.01 mol L^−1^), and the standards solutions were diluted in water.

## 3. Results and Discussion

### 3.1. Parameters Optimization

Initially, we carefully evaluated all the parameters likely to affect the effectiveness of this analytical procedure for the colorimetric determination of calcium in saliva samples with the aim of optimizing them and increase the method’s sensitivity and applicability.

#### 3.1.1. Effect of the Photo-Capture Detector

**Figure 2:**
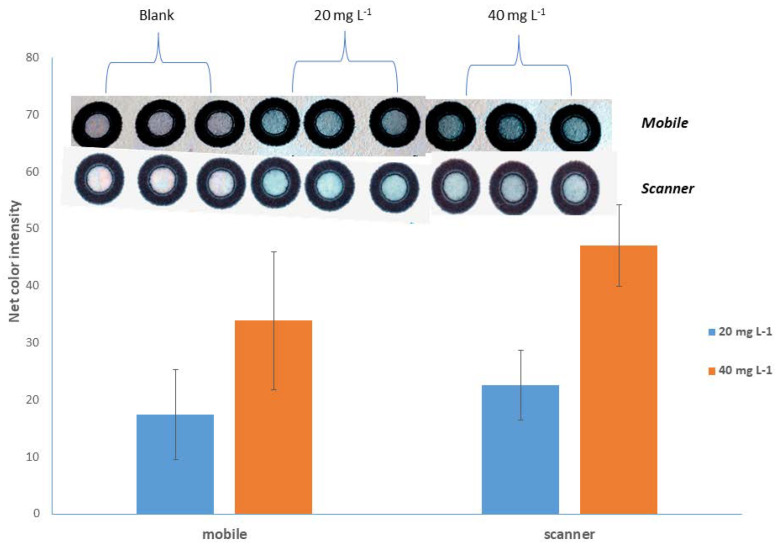
Effect of the photograph-receiving device. Error bars indicate the standard deviation for *n* = 3.

In such instrumentation-free methods, the role of the detector is performed with remarkable success by a domestic photo capture device [44]. Τhe evolution of technology makes it possible to use many and imaging devices with different capabilities, such as digital cameras, smartphones, web cameras, and flatbed scanners. In this method, a domestic flatbed scanner and a camera of a smartphone were used and compared. Standard calcium solutions of 20.0 and 40.0 mg L^−1^ were prepared, the color change in the sensing area with both devices was accomplished, and the mean signals of the color variation for both “detectors” were measured (Figure 2). Motivated by these findings, the use of the scanner was preferred, as it offered (a) higher net value signals (Figure 2), (b) higher signal differences between both standard calcium concentrations, and (c) greater stability in the image capture conditions, because external lighting conditions could not affect the overall process.

#### 3.1.2. Effect of Na_2_SO_3_ Concentration

**Figure 3:**
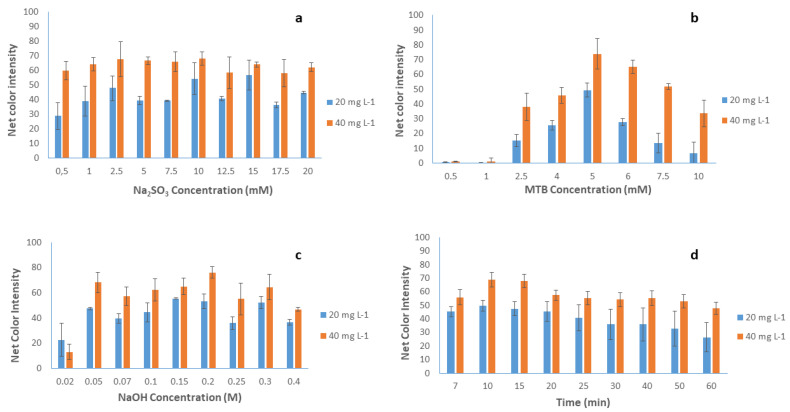
Optimization of (**a**) Na_2_SO_3_ concentration (Ca^2+^ 20 and 40 mg L^−1^, NaOH concentration 0.2 mol L^−1^, MTB concentration 5 mM, reaction time 10 min), (**b**) MTB concentration (Ca^2+^ 20 and 40 mg L^−1^, NaOH concentration 0.2 mol L^−1^, Na_2_SO_3_ concentration 2.5 mM, reaction time 10 min), (**c**) NaOH concentration (Ca^2+^ 20 and 40 mg L^−1^, Na_2_SO_3_ concentration 2.5 mM, MTB concentration 5 mM, reaction time 10 min), and (**d**) reaction time (Ca^2+^ 20 and 40 mg L^−1^, Na_2_SO_3_ concentration 2.5 mM, MTB concentration 5 mM, NaOH concentration 0.2 M). Error bars are the standard deviation for *n* = 3.

The experimental procedure of the proposed method was achieved in ambient conditions, and the paper-based devices were affected by atmospheric air, and since the calcium methylthymol blue complex has a strong tendency to fade due to its oxidation by atmospheric oxygen, the addition of sodium sulfite increased the color stability for up to 12 h [49]. Based on this report, as well as on the optical observation we made during our preliminary experiments, we decided to add sulfite ions to keep the color of the complex stable. Initially, the method was studied with and without the addition of sulfite ions, and it was found that their addition improved the analytical signal by practically tripling it, even at exceptionally low concentrations. The concentration of sodium sulfite was studied in the range from 0.5 to 20.0 mmol L^−1^ by adding 1 μL of the appropriate diluted solution on the paper surface for two different calcium concentrations (20 and 40 mg L^−1^). The experiments revealed a maximum net colorimetric signal, as well as a maximum difference between the two calcium concentrations at a sulfite concentration of 2.5 mmol L^−1^, and thus this concentration was chosen for the subsequent experiments (Figure 3a).

#### 3.1.3. Effect of MTB Concentration

Another important parameter for the complexation reaction between methylthymol blue (MTB) and calcium ions is the concentration of the complexation reagent. Thus, the influence of MTB concentration was studied in an extensive range of concentrations between 0.5 and 10.0 mmol L^−1^, and as a result, the maximum net colorimetric signals were acquired at 5.0 mmol L^−1^ MTB, as shown in Figure 3b. At MTB concentration values lower than the optimum, the formation of the colored complex was not completed, and the color disappeared after a few minutes, while at MTB concentration values higher than 5.0 mmol L^−1^, the excess of MTB turned the color of the sensing area into dark blue and, after the addition of the analyte, there was no significant color difference, which resulted in a significantly lower intensity variation between the blank and the sample signals.

#### 3.1.4. Effect of Sodium Hydroxide Concentration

The formation of the calcium–MTB complex has been studied in detail, and the experimental literature reports agree that the formation of the complex takes place under high alkaline conditions, at pH values higher than 11 [36,49]. The influence of NaOH concentration on the evolution of the complexation reaction was studied in the NaOH concentration range from 0.02 to 0.4 mol L^−1^ by adding 1 μL of the appropriate diluted solution on the paper surface. The experiments showed that maximum complex formation was achieved for a NaOH concentration of 0.2 mol L^−1^, and thus this concentration was chosen for the subsequent experiments (Figure 3c).

#### 3.1.5. Effect of the Reaction Time

The integration of the complexation reaction and the formation of the colored complex play a catalytic role in this method, in terms of both its accuracy and its reproducibility. Additionally, the paper devices must have enough time to dry and provide reliable results. The color development in aqueous solutions based on the formation of the complex between MTB and calcium is a rapid phenomenon at room temperature [36]. However, due to the different substrate used in the present study (paper devices), it was considered mandatory the study of the reaction time for the quantitative formation of the complex between calcium ions and MTB molecules. Consequently, the effect of the reaction time was studied for various time internals in the range between 7 min (the shortest time necessary in our observations for the analytical paper-based devices to dry) and 60 min, as depicted in Figure 3d. It was apparent that the reception of the photograph should be conducted for a reaction time between 10 and 15 min. For shorter reaction times, the paper substrate did not have enough time to dry, a portion of the reagents remained in the scanner, and the color change was minimized, while for longer reaction times, the color on the paper layouts slowly faded due to oxidation by atmospheric air. Τo make the method faster and since the net signal differences between 10 and 15 min were small, we preferred to use the time of 10 min in all experiments.

#### 3.1.6. Effect of the Detection Zone Size

The size of the detection zone can significantly affect the color development during the complexation process. Since the devices we used were circular, it was considered necessary to study their radius. Exceedingly small paper layouts may increase color intensity, as more reagent molecules are concentrated in a smaller area but may create imperfections when taking the photo (high zoom to measure them), while significantly large detection areas are likely to create problems, such as not filling the entire detection area or the coffee ring effect. Thus, two sizes of medium-radius (relative to detailed paper-based layouts) devices were studied. Specifically, the largest one was a device with a total diameter of 0.7 cm and an inner diameter of 0.5 cm and 0.4 cm (before and after the baking stage of the devices, respectively). The dimensions of the smaller device were 0.6 cm, 0.4 cm, and 0.3 cm, respectively. A standard solution of calcium with a concentration of 20 mg L^−1^, a 0.2 mol L^−1^ NaOH solution, a 2.5 mmol L^−1^ Na_2_SO_3_ solution, and a 5.0 mmol L^−1^ MTB solution were prepared for this experiment, the samples were allowed to dry for 10 min, and then the signal was measured. It was proved that there was no significant difference in the net color values. Specifically, the small device showed a color intensity value of 36.2 ± 1.8, while the large device showed a color intensity value of 36.8 ± 1.3. Therefore, there was no significantly optimal size of detection area, provided that the amounts of reagents were fairly distributed.

#### 3.1.7. Order of the Reagents

Finally, the last parameter studied was the order of the reagents added on the paper surface for the complex formation as well as the calcium concentration determination. Two different reagent orders were studied. In both these procedures, the first reagent added was sodium sulfite to ensure the stability of the formed Ca–MTB complex, and the last reagent added was the analyte (Ca^2+^) to ensure that the device was portable and could be used in situ, with all the appropriate reagents for the analysis preadded in the laboratory. Then, two different experimental routes were followed: (a) addition of NaOH followed by MTB and (b) addition of MTB followed by NaOH. The results when studying this parameter were of significant importance, as the analytical signal was approximately fourfold higher following the first experimental path. This can be attributed to the fact that the analytical apparatus was initially pH-adjusted, and consequently, the addition of the complexing reagent led to the immediate and most efficient formation of the colored complex.

### 3.2. Analytical Characteristics of the Method

Under the optimum experimental conditions, the analytical features of the method were determined in terms of accuracy, linearity, precision, selectivity, and stability.

#### 3.2.1. Linearity, Precision, and Limits of Detection (LOD) and Quantification (LOQ)

The method produced an accumulative (integrating the results from 30 standard solutions analyzed on different working days (*n* = 5)) calibration equation

**Figure 4:**
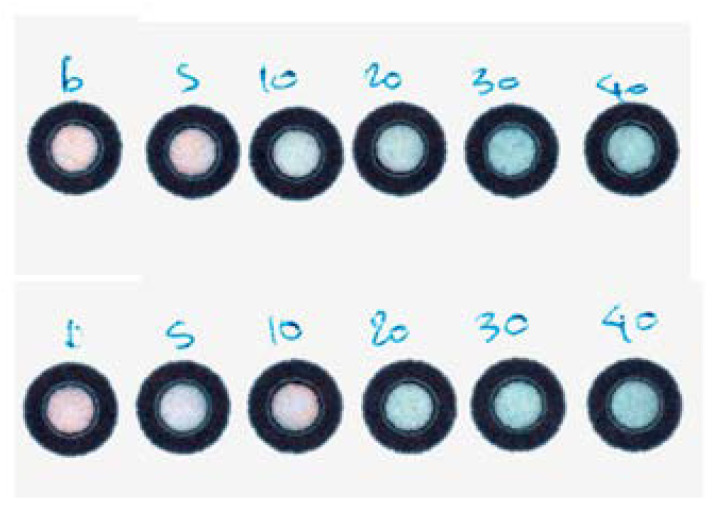
Color alteration for calcium determination according to the method, in the concentration range between 5 and 40 mg L^−1^, visible even to the naked eye.

CI = 1.52 (±0.25) [Ca^2+^] + 14.08 (±1.35), r^2^ = 0.983, (1)

that offered satisfactory linearity in the range between 5 and 40 mg L^−1^ of Ca^2+^, visible even to the naked eye (Figure 4).

In the above Equation, CI is the color intensity measured by the proposed method.

The within-day and intra-day precision was validated for two different calcium concentration values (20.0 and 30.0 mg L^−1^) after repeatable measurements on different paper-based devices (*n* = 5). Additionally, the intra-day relative standard deviation (% RSD) was 5.1% and 4.2% for 20.0 and 30.0 mg L^−1^ concentration levels of the analyte. and the inter-day standard deviation was 5.8% and 6.4%, respectively.

Finally, the Limit of Detection (LOD) as well as the Limit of Quantification (LOQ) were calculated based on the following Equations:

LOD = 3.3 × SD/s and LOQ = 10 × SD/s (2)

where SD is the standard deviation of the intercept, and s is the slope of the respective regression equations. The LOD and LOQ of the proposed method for the analytical determination of Ca^2+^ were 2.9 and 8.9 mg L^−1^, respectively. The analytical characteristics of this method for the determination of calcium in saliva samples resemble in accuracy and reliability those of even standard and well-tested colorimetric methods such as ready-made kits [50]. The detection limits are directly comparable (1.0 mg L^−1^ compared to 2.9 mg L^−1^ for our method), but the stability and the portability of our devices make our method very promising for point-of-need analyses.

#### 3.2.2. Selectivity

Interfering ions, both anions and cations, present in saliva samples were analyzed to determine their possible effect on the analytical signals in the present study. Ions, such as Na^+^, K^+^, Cl^−^, HCO_3_^−^, HPO_4_^2−^ were measured at 100 mmol L^−1^, while Mg^2+^ was measured at 5 mg L^−1^, and Ca^2+^ concentration was fixed at 20 mg L^−1^ in all cases. Mg^2+^ is known to form stable complexes with MTB at an alkaline pH [51], but it has been reported that the calcium concentrations (1.0 to 4.0 mmol L^−1^) are 5–20 times higher than the magnesium ones (0.2 mmol L^−1^) in saliva samples [52]. Thus, the tolerance limit of 5 mg L^−1^ when determining a calcium concentration of 20 mg L^−1^ (ratio 4:1) is considered satisfactory. Additionally, if the measurement of excessively high magnesium/calcium ratios is necessary, in different sample matrices, magnesium can be easily masked with 8-hydroxyquinoline to completely remove it from the sample, without affecting the concentration of calcium [36]. Other co-existing ions in saliva such as SCN^−^ and F^−^ were not studied due to their at least 10–12 times lower concentration compared to calcium, as well as their inability to form complexes with MTB molecules. The experimental results are shown in Table 1 and verified the adequate selectivity of the procedure, considering the expected levels and ratios of Ca^2+^ in the saliva samples.

The influence of all ions was examined at a variety of concentrations starting at a high interferant-to-analyte ratio (500/20 mmol L^−1^), with decreasing concentrations to identify the endurance limit of the method. The concentration of the interfering ions (higher than 100 mmol L^−1^) affected the color on the devices, but their quantity in saliva samples is radically lower, and thus the tolerance limit at 100 mmol L^−1^ was considered satisfactory. Additionally, all different ions revealed no interference for concentrations up to 100 mmol L^−1^, while for higher values of each interferant concentration, the ionic strength as well as the ion charge amount made calcium complexation significantly more incompatible due to competition because of electrostatic charges and thus repulsive forces, especially in samples with water as a solvent.

#### 3.2.3. Stability of the Paper Devices

The main advantage of the paper-based analytical devices is their portability; therefore, it was of immense importance to access their consistency at the point of need. For this purpose, we added solutions of Na_2_SO_3_, NaOH, and MTB (optimum conditions) on the detection zone and we studied the devices’ stability at different temperatures for different time periods. We placed the devices in air-tight bags and kept them for a period of 2, 4, and 6 days in a freezer (−18 °C), a refrigerator (+4 °C), and at room temperature, (+25 °C), always protected from light to avoid the degradation of the reagents. For the determination of calcium, we removed the devices from the freezer and the refrigerator, left them at room temperature for at least one hour, protected from light, and as soon as the devices were equilibrated at ambient temperature, we added the analyte at a concentration of 20 mg L^−1^ Ca^2^. The results for the stability of the devices calculated as % recoveries of Ca^2+^ are summarized in Table 2. The devices were stable for at least 6 days when they were stored at −18 °C without the influence of light.

### 3.3. Application in Real Saliva Samples

Five saliva samples from different adults were measured with the developed PAD method. The samples were treated as described in the Experimental Section. They were diluted 2 to 8 times to be measured within the limits of the calibration curve, and the results can be seen in Table 3. The evaluation of the accuracy of the developed method included the analysis of the same saliva samples by a standard UV–Vis method (see Section 2.6. for experimental details). The normal pH range of saliva is from 6.2 to 7.6, and the experimental conditions for this determination required 0.2 mol L^−1^ NaOH; therefore, it was rather unlikely that the pH of saliva could alter the pH of the devices. The results and the calculated relative errors (%) are summarized in Table 3. The calculated relative errors were between −4.6% and +14.3% for the real samples. This indicated that the average accuracies were satisfactory, and the results of the method developed for calcium determination in saliva samples displayed fair agreement with those obtained with the reference method.

This device showed lower concentration levels for the three of the five samples studied. As it can been noticed in Table 3, the higher the Ca level, the higher the relative error. This can be attributed to interference because of the matrix effect of the saliva samples. The matrix effect tends to add positive errors in analytical methods especially, when analyzing bioanalytical samples such as saliva. Additionally, the low dilution factors used for the proposed determination of calcium in real samples could increase the influence of the matrix effect, in contrast to high dilution factors that can drastically reduce and even eliminate this phenomenon. Finally, the magnitude of these errors is acceptable for bioanalytical applications, in any case.

## 4. Conclusions

This work describes a new paper-based method for the analytical determination of calcium with high selectivity in saliva samples. For this process, we used commercially available reagents. This method is characterized by its low cost, ease of detection, and absence of analytical instrumentation. The selectivity of the method relies on the high ability of calcium to create colored complexes with MTB in alkaline conditions. The signal developed was measured with a flatbed scanner, providing the ability for the quantification of the analyte with minimal treatment at the point of need. The concentration levels of Ca^2+^ in the real samples were confirmed by a UV–Vis method, and the relative errors ranged from −4.6 to +14.3%. Additionally, the method is sensitive for the analysis of Ca^2+^ at low milligram levels (LOD 2.9 mg L^−1^). These findings show the potential of paper-based colorimetric detection in bioanalytical studies.

## Figures and Tables

**Table 1:** Effect of various ions on the determination of calcium (20 mg L^−1^) under the optimum experimental conditions.

Ion Added	Concentration (mmol L^−1^)	Relative Error (%)
Na^+^	100	−11.5
K^+^	100	1.8
Mg^2+^	5 mg L^−1^	2.2
Cl^−^	100	−7.0
HCO_3_^−^	100	−5.0
HPO_4_^2−^	100	−2.3

**Table 2:** Stability of the devices (Na_2_SO_3_ + NaOH + MTB) under different storage conditions.

	Time (days)		
	2	4	6
Temperature (°C)		Recovery %	
25	113.3	92.8	76.4
4	101.6	94.5	84.5
−18	112.2	108.6	114.4

**Table 3:** Comparison of the results obtained with the paper-based method and with the UV–Vis method.

Sample	Calcium Found (mg L^−1^) ^a^		
	UV-Vis	Paper-Based Method	Relative Error (%)
1	57.64	62.12	+7.8
2	54.39	61.01	+12.2
3	73.63	84.15	+14.3
4	44.11	42.98	−2.6
5	32.18	30.71	−4.6

^a^ Mean of three measurements.

## Data Availability

Not applicable.
